# Clarifying comparative claims in digital cognitive assessment: a matters arising on multimodal detection of cognitive impairment and amyloid positivity

**DOI:** 10.1186/s13195-026-02057-w

**Published:** 2026-05-19

**Authors:** Paul W. Estes

**Affiliations:** Cognivue, Inc., Victor, NY USA

**Keywords:** Digital Cognitive Assessment (DCA), Alzheimer’s Disease (AD), Amyloid Biomarkers, Machine Learning, Cognivue, Clock Draw Test

## Abstract

Digital cognitive assessments represent an important and rapidly evolving approach to detecting cognitive impairment and Alzheimer’s disease–related pathology. Jannati et al. report promising findings using a multimodal digital clock and recall assessment to identify cognitive impairment and amyloid positivity. While this contribution is welcomed, several comparative statements regarding Cognivue^®^ rely on historical regulatory data and do not reflect the contemporaneous peer-reviewed evidence base available at the time of manuscript preparation. This Matters Arising article clarifies the limitations of cross-study performance comparisons, addresses selective citation of a single early Cognivue publication, and summarizes subsequent peer-reviewed findings demonstrating Cognivue’s psychometric validity, biomarker associations, and generalizability across diverse populations. These considerations highlight the importance of harmonized, contemporaneous comparisons when asserting relative performance among digital cognitive assessment tools.

## Background and scope

We applaud the work by Jannati et al. describing the Digital Clock and Recall (DCR) test, developed by Linus Health, reporting encouraging results for detecting cognitive impairment and amyloid positivity using multimodal machine-learning models. The study contributes meaningfully to the literature on scalable, technology-enabled cognitive screening. The Bio-Hermes-001 clinical study provides a novel approach to assess the validity, sensitivity, specificity, and predictive values of several digital cognitive assessments with expert clinical diagnoses, plasma biomarkers, and amyloid PET scans. While the report highlighted the DCR findings, it simultaneously compared the performance against some but not all of the measures collected in Bio-Hermes-001, including Cognivue Clarity^®^_,_ an FDA-cleared computerized cognitive screening test [1]. We would like to take the opportunity to clarify some of the findings made by Jannati et al., and place in context with other published studies using the Bio-Hermes-001 data.

### Limitations of methods, analyses, and interpretations

**Fig. 1:**
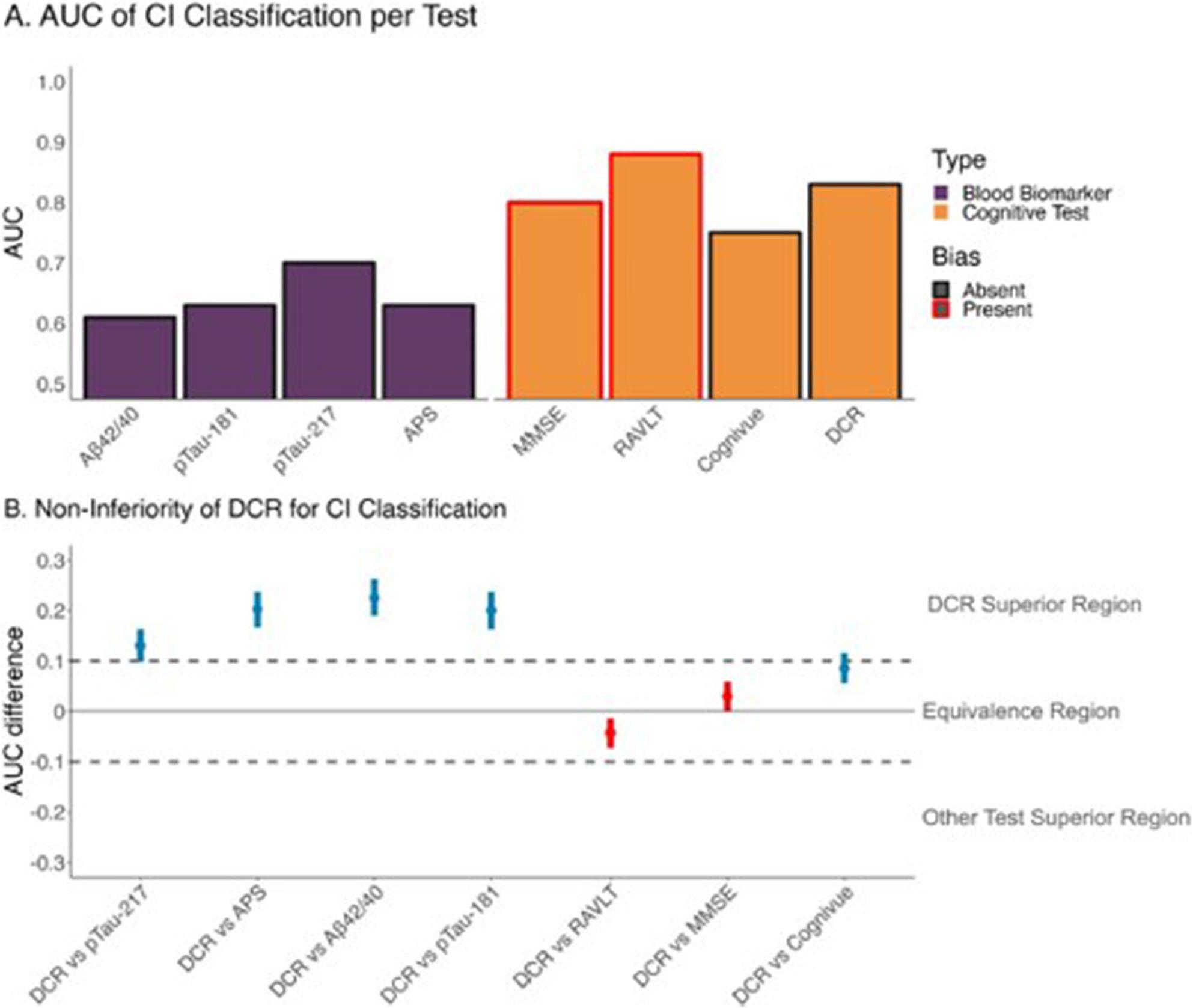
**A** Mean area under the curve (AUC) values (y-axis) averaged across model training iterations for the prediction of cohort classification (cognitively unimpaired [CU] versus MCI or pAD) for each of the cognitive assessments (orange bars) and blood-based biomarkers (BBMs, purple bars). Higher values indicate better performance. Note that the AUC values for MMSE and RAVLT (red bordered bars) were part of the criteria for cohort determination, and their performance Is thus biased. DCR outperformed all other cognitive tests and biomarkers except for the RAVLT, Including the MMSE's biased performance. All BBMs demonstrated poor performance in classifying cognitive impairment. **B** Results of the non-inferiority analysis comparing DCR classification performance against other cognitive tests and B8Ms. Comparisons are listed on the y-axis, while the difference In AUC Is plotted on the x-axis. Median AUC values (blue dots) and the 95% confidence Interval (blue bars) were calculated from 5000 bootstrap iterations. Vertical dashed Iines Indicate the margin=0.1 threshold used to establish non-inferiority/equivalence or superiority of one model's accuracy over another. DCR was statistically superior to all BBMs except for p-tau217 for the classification of cognitive impairment, and marginally superior to Cognitive. Despite the biased performance of the MMSE and RAVLT, the DCR had equivalent performance to those tests for classifying cognitive Impairment

In the Jannati et al. manuscript, the DCR was compared against the Cognivue Clarity and blood-based biomarkers to determine classification of cognitive impairment using a random forest model with “each test’s most predictive variable combined with the participant’s age”. While this approach could work for blood-based biomarkers, we believe this could lead to overfitting the model for Cognivue Clarity since the global scores are already age-normed. Further, in the Non-Inferiority analyses shown in Fig. 1, Panel B, Cognivue Clarity falls within the equivalence region compared to DCR, thus the conclusion that one is superior to the other is not supported by the data. It is also unclear why the analyses were comparing the DCR to the blood-based biomarkers’ predictive ability for detecting cognitive impairment since this is not what the blood-based biomarkers were designed to do, nor what any of them claim.

It is also important to understand what each digital cognitive assessment is actually measuring and how to best interpret their results for use in research and in clinical practice. Is the digital cognitive assessment best for screening for all-cause cognitive impairment? Can it be used for diagnosis of mild cognitive impairment or dementia? How do the scores change over time for tracking progression? Is the digital cognitive assessment sensitive to therapeutic interventions? Understanding these issues can best position the assessments in the most effective, efficient, and appropriate way. This is not discussed in the Jannati et al. manuscript which instead tried to make superiority claims versus Cognivue Clarity and the blood-based biomarkers. However, Cognivue Clarity may fill a different role than DCR, and blood-based biomarkers were never developed to screen for cognitive impairment.

### Contemporary evidence for Cognivue Clarity

Cognivue Clarity was used in Bio-Hermes-001 with three publications reporting the findings. None of these papers were cited in the Jannati et al. manuscript. Specific comparative claims regarding Cognivue Clarity warrant clarification reflecting the value of the Cognivue Clarity. This would also be true for the other digital cognitive assessments included in Bio-Hermes-001 but not examined in the Jannati et al. paper. We have demonstrated that Cognivue Clarity can differentiate between individuals with and without cognitive impairment in the Bio-Hermes-001 sample with large effect sizes and in the biomarker confirmed groups, has a positive likelihood ratio of 2.17, a negative likelihood ratio of 0.29, and a diagnostic odds ratio of 7.48 [2]. Cognivue Clarity was also able to differentiate True Controls (cognitively normal/amyloid negative), Preclinical AD (cognitively normal/amyloid positive) with three subtests being particularly sensitive to the presence of amyloid as defined by PET [3]. Finally, in the same Bio-Hermes sample, we leveraged the three amyloid sensitive tests of Cognivue Clarity to create a Cognivue Amyloid Risk Measure (CARM) using machine-learning to provide added value for the clinician or researcher [4]. The failure to cite contemporary literature using the same cohort is a limitation in fully interpreting the findings of the Jannati et al. report. The assertion that DCR *uniquely* adds predictive value to blood-based biomarkers is difficult to fully evaluate for the reader when findings from other papers published using the same cohort are not provided or discussed.

## Conclusions

Rigorous, transparent evaluation of digital cognitive assessments is essential. This includes testing these assessments in diverse, representative populations and including longitudinal visits to understand their value across the disease spectrum [5]. While “head-to-head” comparisons seemingly provide useful information, they are inherently biased as the methods by which they choose to conduct the comparison are commonly skewed in favor of the author group. This most transparent comparison between two tests would be use of the global scores of the tests that are readily available to clinicians and researchers. That said, Jannati et al. are correct in their assertion that digital cognitive assessments provide a tremendous opportunity for early detection of cognitive impairment in the primary care setting with expedient referral to specialist for formal diagnosis and treatment with current and emerging therapies.

## Data Availability

Data sharing is not applicable to this article as no datasets were generated or analyzed during the current study.

## Abbreviations

AUC: Area under the curve
CARM: Cognivue Amyloid Risk Measure
DCR: Digital Clock and Recall
FDA: Food and Drug Administration
PET: Positron emission tomography
